# The Effect of Long-Term Low-Dose Atropine on Refractive Progression in Myopic Australian School Children

**DOI:** 10.3390/jcm10071444

**Published:** 2021-04-01

**Authors:** William Myles, Catherine Dunlop, Sally A. McFadden

**Affiliations:** College of Engineering, Science and Environment, University of Newcastle, Callaghan, NSW 2308, Australia; c.dunlop8@bigpond.com

**Keywords:** myopia, atropine, 0.01%, eye growth, refractive error, Australia

## Abstract

Myopia will affect half the global population by 2050 and is a leading cause of vision impairment. High-dose atropine slows myopia progression but with undesirable side-effects. Low-dose atropine is an alternative. We report the effects of 0.01% or 0.005% atropine eye drops on myopia progression in 13 Australian children aged between 2 and 18 years and observed for 2 years without and up to 5 years (mean 2.8 years) with treatment. Prior to treatment, myopia progression was either ‘slow’ (more positive than −0.5 D/year; mean −0.19 D/year) or ‘fast’ (more negative than −0.5 D/year; mean −1.01 D/year). Atropine reduced myopic progression rates (slow: −0.07 D/year, fast: −0.25 D/year, combined: before: −0.74, during: −0.18 D/year, *p* = 0.03). Rebound occurred in 3/4 eyes that ceased atropine. Atropine halved axial growth in the ‘Slow’ group relative to an age-matched model of untreated myopes (0.098 vs. 0.196 mm/year, *p* < 0.001) but was double that in emmetropes (0.051 mm/year, *p* < 0.01). Atropine did not slow axial growth in ‘fast’ progressors compared to the age-matched untreated myope model (0.265 vs. 0.245 mm/year, *p* = 0.754, Power = 0.8). Adverse effects (69% of patients) included dilated pupils (6/13) more common in children with blue eyes (5/7, *p* = 0.04). Low-dose atropine could not remove initial myopia offsets suggesting treatment should commence in at-risk children as young as possible.

## 1. Introduction

Myopia (short-sightedness) is the leading form of refractive error, whereby the retinal image is focused in front of the retina. The incidence of myopia is increasing worldwide with half the global population predicted to be affected by 2050 [1]. High myopia, defined as a refractive error more than −6.0 diopters (D) or ocular axial length of more than 26–26.5 mm [2], is associated with an increased risk of developing vision-threatening retinopathies including retinal detachment, choroidal neovascularisation, chorioretinal atrophy and macular atrophy [3,4]. Single vision corrective lenses can help restore vision at distance, however they do not halt the progression of myopic eye growth.

Muscarinic receptor antagonists such as atropine are currently the most effective pharmacological interventions for slowing (but not eliminating) both the advancement of refractive error and axial growth [5]. The use of high concentration atropine eye-drops (0.5–1.0%) for the treatment of myopia is well documented [6,7]. More recently, the prescription of low-dose atropine eye-drop preparations (0.01%) for the treatment of myopia in children has gained popularity [8,9,10,11,12]. Low-dose atropine has several advantages over higher concentrations including lower rebound axial growth and refractive error following treatment cessation and lower incidences of side-effects such as allergic reactions, glare and near visual loss compared with higher concentration preparations whilst still showing efficacy in slowing the progression of myopia [9,10,13,14,15]. Although encouraging, these results represent a majority Asian [8,10,11,13] (67%) and Spanish [12] (15%) cohorts with <18% Caucasian participants [11,14,15], for which no data exceeding one year of treatment is available. It is also well known that Atropine binds to pigment in the iris and would cause different release properties in dark versus pale coloured eyes [16]. We present here a small retrospective study into a relatively long-running data set of Australian school children receiving low-dose atropine (0.005–0.01%) for the treatment of myopia.

## 2. Experimental Section

### 2.1. Subjects

The present study was conducted as a retrospective analysis of 13 myopic Australian children between 2 and 18 years of age prescribed low-dose atropine for the treatment of myopia in subjects referred to an Ophthalmology clinic in Newcastle, NSW, Australia. Patients with a minimum of −0.25D refractive error in one eye at the start were included in the present study. Informed consent for analysis of the data was obtained from a parent or guardian of the children. Half the patients were male and half female. Half the participants had at least one parent with myopia (Table 1). All patients wore corrective eyeglasses, except one patient who had been prescribed hard contact lenses. This latter patient was excluded from analyses of refractive error and ocular measurements but was included for analyses relating to eye colour and adverse side effects of atropine treatment.

### 2.2. Procedure

Measures were taken on average every 9 months and timing varied between patients (Table 1). Patients’ refractive error was monitored for an average of 2 years (maximum of 7 years, Table 1) prior to treatment. Treatment lasted on average 3 years (maximum of 5 years, Table 1), and initially consisted of atropine sulphate monohydrate eye drops (0.01% in saline, diluted from commercially available stock solution: Atropt 1%, Aspen Pharma Pty Ltd., Dandenong, Australia) to be self-administered once daily in the evenings, in addition to corrective eyeglasses. Note, Atropt 1% solution contained the preservative benzalkonium chloride. Guardians and patients were asked about their compliance with the daily protocol in regular visits to the clinic. Any patient experiencing persistent ocular discomfort from 0.01% atropine was given the option to have their prescribed dose halved to 0.005% (single use vials, Atropt 1%, Aspen Pharma Pty Ltd., Dandenong, Australia). At each regular visit, measures were taken of refractive error, axial length and cornea power in both eyes.

### 2.3. Ocular Measures

Refractive error was determined using streak retinoscopy after induction of cycloplegia using 1% cyclopentolate hydrochloride (Cyclogyl eye drops 1.0%, Alcon Inc, Geneva, Switzerland). Axial length and cornea power (K1, K2) were measured using a Zeiss IOLMaster^®^ 500 (Carl Zeiss Meditec Inc., Dublin, CA, Ireland). The average of five readings for axial length and the average of three readings for corneal curvature were taken for analysis. Corneal Power K was calculated as the average of K1 and K2.

### 2.4. Analyses

Patients were separated into two groups based on the rate of their myopia progression (change in spherical equivalent over time) with and without atropine being less than or equal to 1.5 standard deviations from the mean of the group. Two participants did not have a recorded baseline rate of myopia progression, so only the rate during atropine treatment was used. (These two participants were excluded from comparisons of the effect of atropine on refractive error progression rates). This resulted in two distinct groups of myopes who could be defined as having either a pre-treatment myopia progression rate of more positive than −0.5 D per year, termed the ‘slow’ progressing group, or a pre-treatment myopia progression equally or more negative than −0.5D per year, termed ‘fast’ progressing group. There were no subjects with a progression rate equal to −0.5 D/year. One notable outlier was classed as ‘Ultrafast’ as they exhibited a rate of refractive error change during treatment that was >3 standard deviations from the mean of the fast progressing group. Note that these group segregations were based on refractive error progression rates, not axial elongation rate.

**Figure 1:**
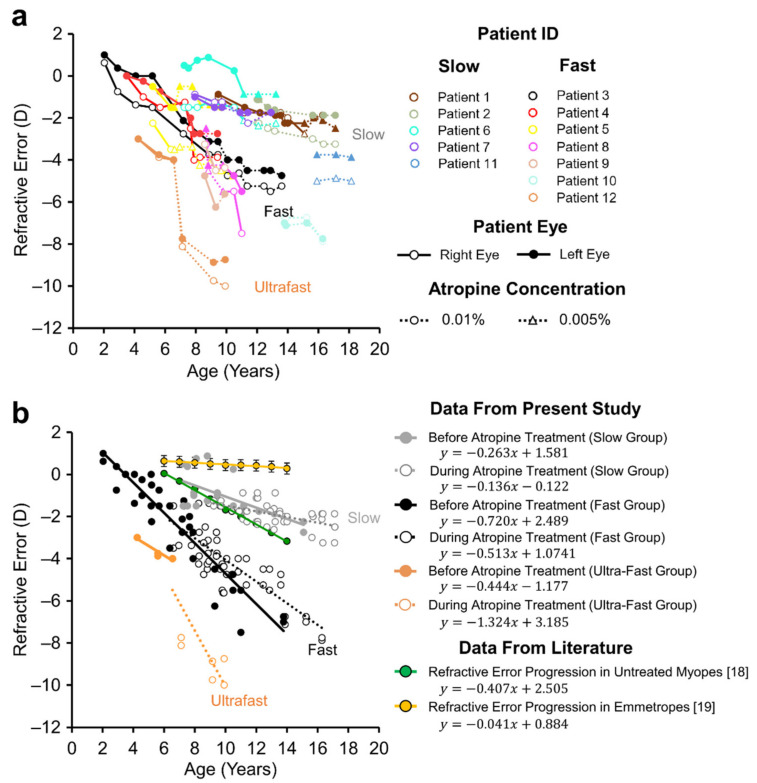
Patient refractive error prior to and during low-dose atropine treatment. (**a**) Individual patient refractive error prior to atropine treatment (solid lines) and during atropine treatment (dotted lines) reported for both right (open markers) and left eyes (closed markers). Triangle markers indicate 0.005% Atropine for patients 1, 5, 6, 8 and 11 (**b**) Patients were segregated into slow (depicted by grey markers) and fast (depicted by black markers) progressing myopes based on the rate of refractive error advancement prior to treatment. Linear fits were applied to untreated (solid lines) and atropine treated (dotted lines) data points. Refractive error data from untreated myopes (green) and normal emmetropes (yellow) obtained from the literature are included for comparison.

Statistical analyses used IBM^®^ SPSS^®^ Statistics, Version 24 (IBM, Armonk NY, USA). Seven patients across both the fast and slow groups were used for statistical comparison of refractive error progression before and after atropine treatment. Four patients (numbers 2, 9, 10, 11 in Figure 1a) with insufficient observation time (less than six months) before or after receiving treatment were excluded from this comparison. The ultrafast progressing Patient 12 was also excluded. The rate of progression in refractive error prior to treatment was based on linear regression fits over the pre-treatment period excluding subjects in which the observation time was less than six months. During atropine treatment, the rate of progression in myopia was based on linear regression fits from the commencement of atropine treatment to the last visit in which treatment compliance had been confirmed, and thus were calculated over the entire course of treatment to date (this method was also used when calculated the rate of axial length growth). Data points collected prior to 5–6 years of age were excluded from the fits as they would likely include an expected emmetropisation based reduction in rate of refractive error change over this time period [17]. A 2 × 2 model repeated measures ANOVA was used with individual eye measurements and treatment included as within-subject variables.

To determine whether low-dose atropine influenced axial length growth, eye growth data collected during treatment was compared to age matched data from the published literature [18,19]. These two studies were selected because of the strength of their study design and the use of Caucasian subjects making comparison with our Australian subjects potentially more relevant. Specifically, the rate of growth was calculated for each patient’s eye and then compared with the predicted growth at the same age and over the same duration from models of growth in normal emmetropic school children [18] (*n* = 194, age 6–14, Equations (1) and (2)), and in untreated myopes [18] (*n* = 247, mean baseline age 7.98 ± 2.1, Equations (3) and (4)). The models used were as follows.

Model of axial length growth in emmetropic school children [19] when:

Age was ≤10.5 years:

Axial Length (mm) = 17.808 + 2.560 · ln(age),(1)

Age was >10.5 years:

Axial Length (mm) = 18.144 + 2.391 · ln(age),(2)

Model of axial length growth in untreated myopes [18] when:

Age was ≤10.5 years:

Axial Length (mm) = 20.189 + 1.258 · ln(age),(3)

Age was >10.5 years:

Axial Length (mm) = 21.353 + 0.759 · ln(age),(4)

Paired *t*-tests or related-samples Wilcoxon signed rank tests were used to determine whether the rate of axial length change observed in myopes treated with low-dose atropine were significantly different to rates predicted in untreated myopes and emmetropes.

Two-way mixed model ANOVA with individual eye measurements included as a repeated measure were used to compare cornea power and the rate of axial growth between slow and fast progressing myopes. In cases where there was a significant difference in the age at measurement, age was included as a covariate. Wilcoxon signed rank tests were used to compare the median rate of refractive error change to that of a published model of untreated myopes [18]. Fisher’s exact tests were used to determine potential effects of eye pigment on the incidence of ocular side-effects associated with atropine. Pearson’s correlation coefficient was used for tests of correlation.

## 3. Results

### 3.1. Daily Low-Dose Atropine Reduced the Progression Rate of Myopia

Two distinct progression rates in refractive error were observed prior to treatment (Figure 1a). Untreated myopes (solid lines in Figure 1a) with ‘slow’ and ‘fast’ progressing myopia had a mean rate of refractive error progression of −0.19 ± 0.14 D/year (*n* = 4) and −1.01 ± 0.56 D/year (*n* = 5), respectively, a five-fold difference (t = 3.459, *p* < 0.011, 2-tailed, Power = 0.85). During treatment with atropine (dotted lines in Figure 1a), the mean rate of refractive error progression in all 9 subjects was −0.09 D/year in the ‘slow’ progressing group and −0.27 D/year in the ‘fast’ progressing cohort.

**Figure 2:**
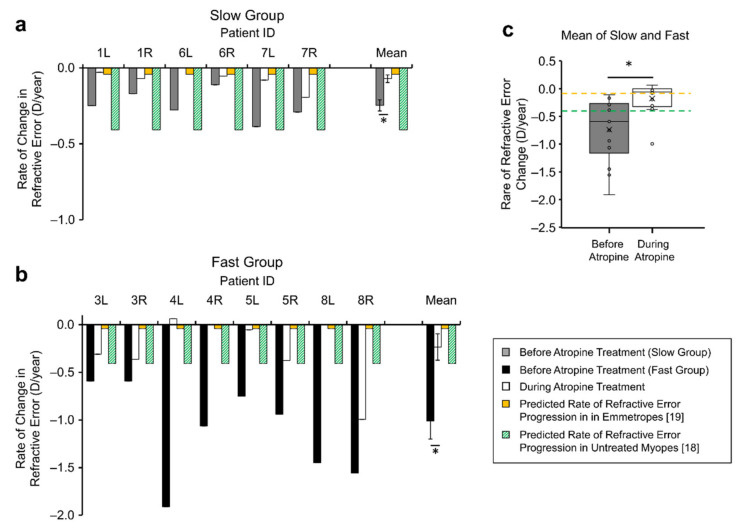
Refractive error progression rates in: (**a**) Slow-progressing myopes, and (**b**) Fast-progressing myopes. (**a**,**b**) show the individual rates of change in refractive error before atropine (solid bars) and during atropine treatment (open bars) for patients with greater than six months of observation time. The predicted rate of change in untreated myopes (green bars) and emmetropes (yellow bars) are included from the literature for comparison. (**a**,**b**) show the corresponding mean rates of change in refractive error for either group and (**c**) shows the corresponding mean rate of change in refractive error for patients from both slow and fast groups, before and after atropine treatment, * *p* < 0.05.

The mean refractive error progression in the seven patients with at least six-months observation time prior to treatment was significantly reduced during atropine treatment (before vs. during: −0.74 ± 0.57 D/year vs. −0.18 ± 0.28 D/year, F = 8.014, *n* = 7, *p* = 0.030, Power = 0.68, Figure 2c). Amongst the included slow progressing myopes, the rate of refractive error change significantly decreased in all eyes (6/6) during atropine treatment (−0.25 ± 0.10D/year vs. −0.07 ± 0.07D/year, Figure 2a, T = −4.088, *n* (eyes) = 6, 2-tailed *p* < 0.01, Table 2). This was also true for fast progressing myopes, with (8/8) eyes showing a reduction in the rate of refractive error change during atropine treatment (−1.10 ± 0.49D/year vs. −0.25 ± 0.35D/year, Figure 2b, T = −3.987, *n* (eyes) = 8, *p* <0.01, Table 2).

The rate of refractive error progression prior to atropine treatment was not significantly different than that predicted by the reference model of untreated myopes [18] (observed median = −0.59, hypothetical median =−0.41, W =−1.014, 2-sided, *p* = 0.310, Power = 0.30), however the rate of refractive error progression during atropine treatment was significantly less than that predicted by the same model of untreated myopia (observed median = −0.06, hypothetical median = −0.41, W = 2.419, 2-sided *p* = 0.016, Power > 0.99, green bars Figure 2a,b).

It was observed that two patients who temporarily ceased atropine treatment experienced a return to their original rate of refractive error progression in 3 out of 4 eyes (patients 1 and 8 in Figure 1, −0.51 ± 0.14 D/year during atropine treatment vs. −1.12 ± 0.28 D/year after cessation, *n* = 3).

### 3.2. Axial Length in Myopes during Low- Dose Atropine Treatment

**Figure 3:**
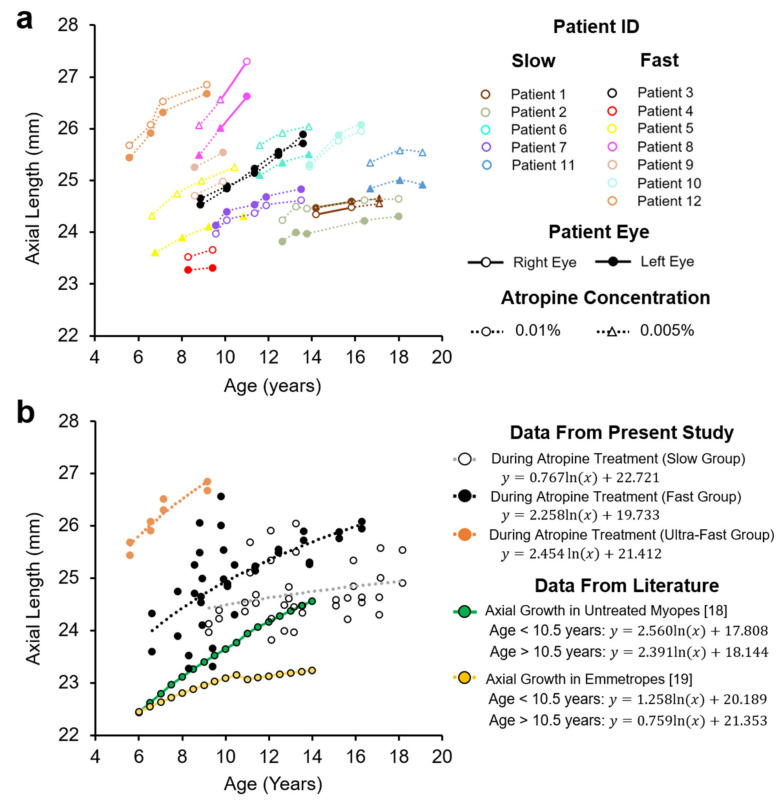
Axial length in patients receiving low-dose atropine. (**a**) Axial length growth in each eye in 12 patients receiving low-dose atropine. (**b**) Axial length data from the present study divided into those with ‘slow’ (unfilled grey markers), ‘fast’ (filled black markers) or ‘ultrafast’ (filled orange markers) progressing refractive errors. Axial length data from untreated myopes and emmetropes from published literature are included for comparison [18,19].

**Figure 4:**
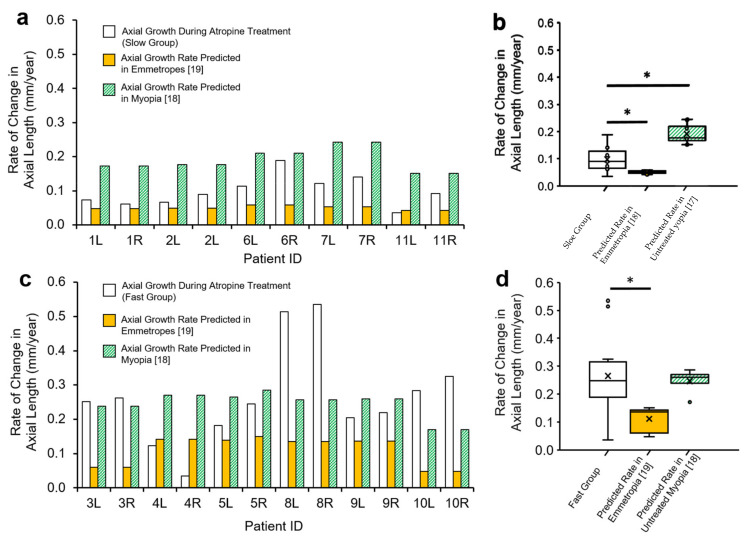
Rate of axial length change in patients receiving low-dose atropine. (**a**) Rate of axial length growth in slow progressing myopes (measurements for individual eyes presented) prescribed low-dose atropine compared with equivalent growth rates over the same age range as predicted in untreated myopes [18] and emmetropes [18,19]. (**b**) Box-plots summarising data in part (**a**). (**c**) Rate of axial length growth in fast progressing myopes prescribed low-dose atropine compared with equivalent growth rates at same age as predicted in untreated myopia and emmetropia. (**d**) Box-plots summarising data in part (**c**), * *p* ≤ 0.05.

Patient axial lengths are shown in Figure 3a. Patients with ‘slow’ progressing myopia receiving low-dose atropine displayed axial length growth that was significantly slower than that predicted in untreated myopes over the same age range [18] (0.098 ± 0.043 vs. 0.196 ± 0.041 mm/year, T = −16.753, *n* (eyes) = 10, *p* < 0.001, Power > 0.99, Figure 4b), but significantly faster than that predicted in emmetropes of the same age [19] (0.098 ± 0.043 vs. 0.051 ± 0.005 mm/year, Figure 4b, W = −2.701, *n* (eyes) = 10, *p* = 0.007, Power = 0.95).

In contrast, patients with ‘fast’ progressing myopia receiving atropine treatment did not show slowed axial growth, being not significantly different on average to growth predicted in untreated myopes matched in age (0.265 ± 0.143 vs. 0.245 ± 0.037 mm/year, Figure 4d, W = −0.314, *n* (eyes) = 12, Power = 0.52, *p* = 0.754). ‘Fast’ progressing myopes with atropine treatment also had axial growth rates that were 2.4 times faster than that predicted in age-matched emmetropes (0.265 ± 0.143 vs. 0.111 ± 0.043 mm/year, Figure 4d, W = −2.589, *n* (eyes) = 12, *p* = 0.010, Power = 0.69). These data are summarized in Table 3.

When our two groups were combined, 16 out of 22 eyes receiving low-dose atropine (73%) had an axial growth rate less than what was observed in untreated myopes, however this did not reach statistical significance (W = 1.477, *n* = 22, 1-sided *p* = 0.07, Power = 0.29).

### 3.3. Axial Length Largely Accounted for Refractive Development

**Figure 5:**
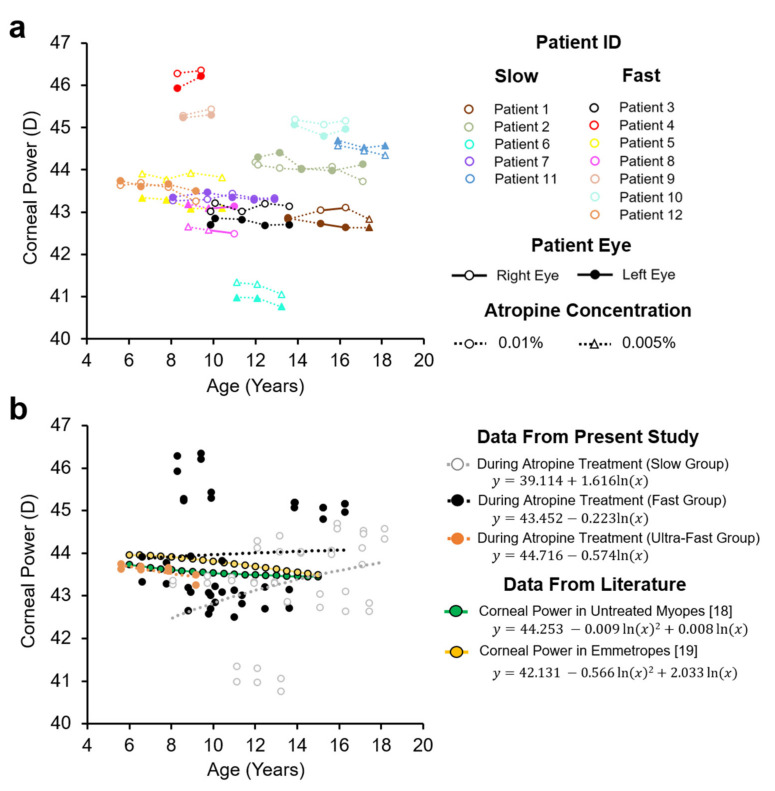
Corneal Power in patients receiving low-dose atropine. (**a**) Change in corneal power in patients receiving low-dose atropine. (**b**) Corneal power data from the present study was separated into those with ‘slow’ (unfilled grey markers), ‘fast’ (filled black markers) or ‘ultrafast’ (filled orange markers) progressing refractive error prior to treatment as defined in Figure 1. Corneal power in untreated myopes and emmetropes are included for comparison [18,19].

Patient corneal power declined with age in 16 out of 24 eyes, however across all patients the mean decline was not significant (−0.02 ± 0.09 D/year, T = −1.284, 1-sided *p* = 0.106) (Figure 5a). There was no statistical difference between the fast and slow progressing groups in their corneal power after accounting for variation in age at their latest visit (43.90 ± 1.73 vs. 44.46 ± 1.70 D, F = 1.789, *n* = 11, *p* = 0.218, Power = 0.26).

**Figure 6:**
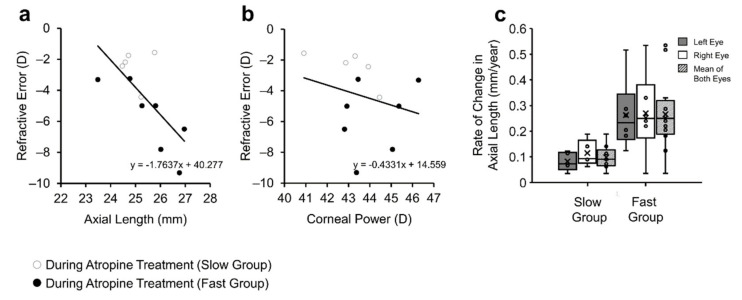
Relationship between Refractive Error and ocular parameters. (**a**) Negative correlation between axial length and refractive error measured during the latest visit. (**b**) No significant relationship between refractive error and corneal power measured during the latest visit. (**a**,**b**) contains data from the right eye only, segregated based on the rate of refractive error change, i.e., ‘slow’ progressing myopia or ‘fast’ progressing myopia (**c**).

Patient refractive error was negatively correlated with axial length measured at their latest visit (r = −0.713, *n* = 12, *p* < 0.01, Figure 6a), but was not correlated with corneal power (r = −0.248, *n* = 12, *p* = 0.437, Figure 6b). The rate of axial elongation during atropine treatment was not significantly different between ‘fast’ and ‘slow’ progressing myopes after accounting for variation in age at the start of atropine treatment (0.265 ± 0.143 vs. 0.098 ± 0.043 mm/year, F = 3.839, *n* = 11, *p* = 0.086, Power = 0.41, Figure 6c).

### 3.4. Adverse Effects of Low-Dose Atropine Treatment

Of the 13 patients who received atropine treatment only four (31%) experienced no side-effects (Table 4). The most common reported side effects of the atropine treatment were dilated pupils (six cases) and dry or irritated eyes (five cases). There was one case of sensitivity to light and two cases of headaches. Of those patients who were experiencing side-effects, five chose to reduce their atropine eye drop prescription from 0.01% to 0.005% (specifically Patients 1, 5, 6, 8, 11, see triangles in Figure 1) after which only one patient (#1) continued to experience issues with pupil dilation. Pupil dilation was more common in children with blue eyes, with five out of seven children (71%) compared to only one patient with non-blue eyes (16.7%) experiencing dilation during treatment (Table 4, *n* = 13, 1-sided *p* = 0.043). There was no significant association between eye colour and any other observed side-effect in this small sample (Table 4).

Three patients (23%) ceased low-dose atropine treatment due to side effects. One patient experienced frequent irritation and headaches, another experienced irritation and pupil dilation and the last patient discontinued treatment because of photophobia which caused them to squint in sunlight.

## 4. Discussion

### 4.1. Low-Dose Atropine for the Treatment of Myopia

**Figure 7:**
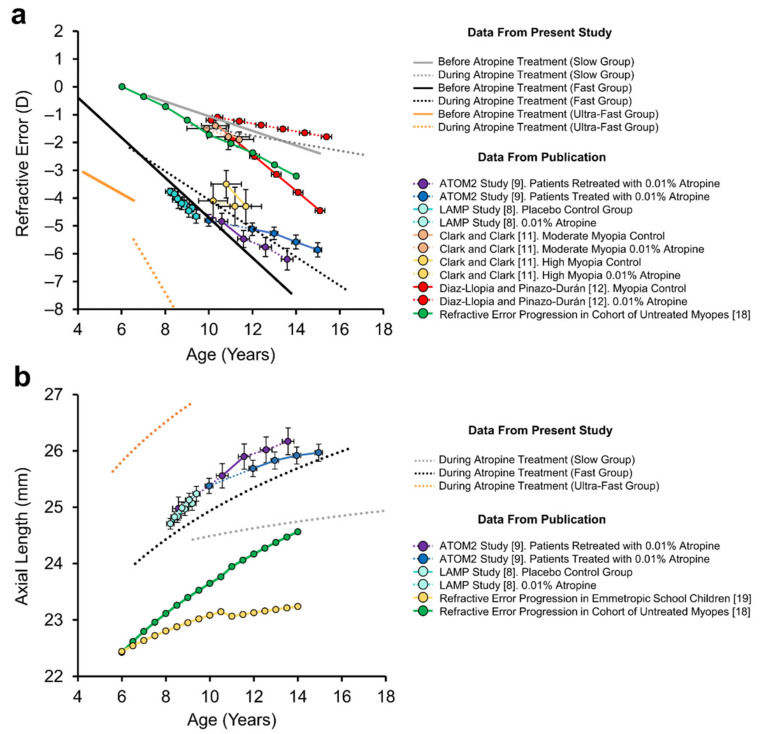
Comparison of present study results with the published literature. (**a**) Fitted regressions from current study (see individual data in Figure 1b) for patient refractive error for ‘slow’ (in grey), ‘fast’ (in black) and ‘ultrafast’ (in orange) progressing myopia groups prior to (solid lines) and during low-dose atropine treatment (dotted lines). Refractive error data from patients receiving low-dose atropine (0.01%) are included from the literature for comparison (coloured solid lines, controls; dotted lines, during atropine treatment). (**b**) Corresponding axial length data from current study (see individual data in Figure 3b) for ‘slow’, ‘fast’ and ‘ultrafast’ progressing myopes receiving low-dose atropine (dotted lines) compared with axial length data from the literature for patients receiving low-dose atropine (0.01%) and age-matched untreated controls (solid lines).

Patients receiving low-dose atropine exhibited on average a 75% reduction in the rate of refractive error progression. This large reduction in refractive error rate is likely exaggerated due to our small sample size and the relatively long duration of observation. Since measurements collected prior to 5−6 years of age would include an expected emmetropisation based reduction in rate of refractive error change [17], only data points after this age range were included in our analysis to ameliorate the effects of emmetropisation on the rate of refractive error change. Ultimately, these factors could account for why the change observed here was greater than the 27% and 38% reductions observed over one and five years of treatment in the LAMP [8] and ATOM2 [9] studies, respectively (See Table 5). However, these data may also suggest a difference in the efficacy of low-dose atropine or higher instances of non-responders to atropine treatment amongst different ethnic populations. Encouragingly, our results were similar to observations made in German [14] and Italian [15] school children who exhibited a 62% and 55% reduction after one year of treatment, respectively (compared within subjects), and were the same as the 75% reduction in refractive error rate observed by Clark and Clark [11] in their ‘higher myopes’ category and the 77% reduction observed by Diaz-Llopis and Pinazo-Durán [12] in Spanish school children (compared to untreated control myopes) (Table 5). Mean refractive error trajectory for each of these studies are included alongside our data for comparison in Figure 7.

### 4.2. Effects of Low-Dose Atropine on Axial Length Progression Rates

The rate of axial length growth in ‘slow’ progressing myopes receiving atropine was half that observed in untreated myopia, however ‘fast’ progressing subjects showed no significant decrease compared to untreated myopes. Importantly, even in our slow progressing group, the rate of axial growth was significantly higher (92%) than values expected in normal emmetropia. This suggests that low-dose atropine does not reverse axial growth in these individuals but reduces it by approximately 50% relative to that observed in untreated myopes, a rate which was still higher than typically observed in emmetropia. It’s important to note that patient axial length remained permanently offset by their existing excess growth prior to treatment. This offset likely results from a period of accelerated axial elongation which occurs prior to myopia onset [21,22]. Given the young age of onset of some of our patients (notably patients 3 and 4, Figure 1), this suggests that excessive axial growth can occur at a very young age and implies that children should receive atropine treatment as young as possible to reduce this offset.

The rate of axial length growth in ‘fast’ progressing myopes receiving low-dose atropine was 2.4× that observed in emmetropia. Although axial length growth in this group was not significantly different to growth in a model of untreated myopes [18], it is important to note that prior to treatment, myopes enrolled in the present study in the fast group displayed greater mean refractive errors and rates of refractive error advancement than those enrolled in Jones’ study [18] (Figure 1b). As such, it could be seen as a positive that axial length growth in children with rapidly advancing myopia was similar to that seen in less severe myopes. For context, axial length data from our ‘fast’ progressing myopia group aligned well with data reported in the ATOM2 and LAMP studies [8,13] (Figure 7b).

### 4.3. Side-Effects of Atropine Treatment

Low-dose atropine is gaining favour due to the low incidence of side-effects observed in cohorts of Asian school children [8,10]. In the present study, results were positive where only one patient experienced headaches associated with the use of atropine drops and only one patient experienced photophobia. Minor side-effects including pupil dilation and irritation were more common. It is known that iris pigmentation can affect the susceptibility of the pupil to atropine induced mydriasis (dilation) [16] which could account for the over-representation of pupil dilation observed in blue eyed children in this study. Loughman and Flitcroft have previously postulated that the side-effects of atropine may be more severe in light-eyed Caucasian patients and investigated the tolerability of 0.01% atropine in university students over the course of five days [23]. They reported that although pupil size and responsiveness was significantly affected, the treatment was generally ‘tolerable’. However, the aforementioned study took place over a short duration and was conducted in university students who would likely be more tolerant of ocular discomfort than children as young as six receiving atropine treatment. These minor side-effects may be especially distressing for parents when individual improvements in myopia progression associated with atropine treatment are subtle.

### 4.4. Limitations

There were clear limitations of the present study. These include the lack of pre-data baseline for axial length (although this was available for refractive error) and the small number of individual patients. These issues reflect problems inherent in a retrospective long-running data set. In several instances, patients were referred to the clinic to receive low-dose atropine treatment, limiting the pre-treatment screening time and as such, the amount of data recorded prior to atropine treatment. As a result, only seven participants with >6 months of observation time prior to treatment were included in our direct comparison of refractive error rate before and after atropine treatment. However, by comparing measurements from both left and right eyes, before and after atropine treatment, we were able to achieve a reasonable statistical power of 0.681. As mentioned previously, an inherent limitation using a repeat measures comparison for comparing the rate of change in refractive error is the potential effect of emmetropisation, particularly prior to 5–6 years of age. We addressed this by excluding data points during this period and by including an additional comparison to an age-matched data set of non-treated control myopes. Never-the-less the data is of interest to report given the considerable period in which some of the children have been under treatment, and relatively limited information available regarding non-Asian cohorts.

## 5. Conclusions

The current study suggests that low-dose atropine (0.01%) may reduce the rate of refractive error progression in myopic children if given as daily eye drops by as much as 75%, but caution is warranted since such changes were observed to rebound when treatment was discontinued. Furthermore, low-dose atropine did not reduce axial growth to normal eye lengths even after up to 5 years of treatment, but for low myopes it may offer some protective effects. Importantly, the data suggests that enhanced eye elongation occurs at very young ages and cannot be undone with low-dose atropine and speaks to the probability that such treatments may need to be applied from a very young age to see truly beneficial effects.

## Figures and Tables

**Table 1:** Patient Characteristics.

Parameters		All	Slow	Fast	*p*-Value
Number of Patients	N	12	5	7	
Gender M/F	N/total	6/6	2/3	4/3	
At least 1 parent with myopia	N/total	6/12	3/5	3/7	
Age at Referral (years)	Mean	7.9 ± 4.2	10.5 ± 3.5	6.6 ± 4.0	0.11
	Range	2.0–15.8	7.25–15.8	2–13.8	
Observation time prior to treatment (years)	Mean	2.0 ± 2.3	1.9 ± 2.0	2.1 ± 2.7	0.89
	Range	0–6.8	0–4.1	0–6.8	
Age at Start of treatment (years)	Mean	10.3 ± 3.0	12.4 ± 2.5	8.8 ± 2.5	0.03
	Range	6.6–15.8	8.7–15.8	6.6–13.9	
Observation time during treatment (years)	Mean	2.8 ± 1.3	3.2 ± 1.2	2.5 ± 1.4	0.28
	Range	1.1–5.0	2.1–5.0	1.1–4.7	
Time between visits and measures (months)	Mean	9.8 ± 6.0	10.1 ± 6.4	9.6 ± 5.8	0.62
	Range	0.7–29.7	1.2–29.7	0.7–24.4	

The speed of progression was based on the progression rate in refractive error prior to treatment. Slow and fast progressing myopia is defined in the Methods. Range and mean time periods ± SD are indicated. N, number, *p*-value (2-tailed) is the difference between slow and fast progressing myopic sub-groups.

**Table 2:** Rate of Change in Refractive Error (D/year) in current study compared to published data for emmetropes and untreated myopes.

Group	Eyes (N)	Before Atropine	During Atropine	*p* Value (Before vs. During)	Emme-Tropia	Untreated Myopia	*p* Value
Slow+ Fast	22		−0.16 ± 0.24		−0.04	−0.41	
	7 ^†^	−0.74 ± 0.57	−0.18 ± 0.28	0.03	−0.04	−0.41	0.31 * 0.02 **
Slow	3 ^†^	−0.25 ± 0.10	−0.07 ± 0.07	<0.01	−0.04	−0.41	
Fast	4 ^†^	−1.10 ± 0.49	−0.25 ± 0.35	<0.01	−0.04	−0.41	

Refraction data from a linear fit. Data presented as mean ± SD. Comparison of current data to published control data: * Comparison between before atropine and untreated myopia, ** Comparison between during atropine and untreated myopia. ^†^ Number of Participants that met the minimum six-month baseline observation period.

**Table 3:** Rate of Change in Axial Length (mm/year) in current study compared to published data for emmetropes and untreated myopes.

Group	Eyes (N)	During Atropine	Emmetropia	Untreated Myopia	*p* Value
Slow + Fast	22	0.191 ± 0.136	0.084 ± 0.045	0.220 ± 0.045	0.07 *
Slow	10	0.098 ± 0.043	0.051 ± 0.005	0.196 ± 0.041	<0.01 * <0.01 **
Fast	12	0.265 ± 0.143	0.111 ± 0.043	0.245 ± 0.037	0.75 * 0.01 **

Axial length data is from a logarithmic fit. Data presented as mean ± SD. * Axial rate during atropine compared with untreated myopia, ** Axial rate during atropine compared with emmetropia.

**Table 4:** Adverse treatments effects of low-dose atropine.

Eye Colour	Patients (n)	Dilated Pupils	Photophobia	Irritation	Headaches	Side- Effects
All	13	6/13 (46%)	1/13 (8%)	4/13 (31%)	1/13 (8%)	9/13 (69%)
Blue	7	5/7 (71%)	1/7 (14%)	2/7 (29%)	0	5/7 (83%)
Brown	4	0	0	2/4 (50%)	1/4 (25%)	2/4 (50%)
Hazel	2	1/2 (50%)	0	0	0	1/2 (50%)
Fisher’s Exact Test *p*-value	13	0.043		0.296		0.391

Effect of eye pigment on incidence of adverse treatment effects tested using Fisher’s exact test.

**Table 5:** Sample details of published studies on low-dose atropine.

Study	Geographic Location	Age Range (years)	Study Duration (years)	Atropine Dose (%)	N (Final)	Inclusion Criteria	RE Rate Change (0.01%)
Dan-Ning Hu, 1998 [20]	China	9–18	1	1, 0.1, 0.01	536	−0.5 D < S.E < −3.0 D	26%
Chia et al., 2016 [9]	Singapore	6–12	5	0.5, 0.1, 0.01	345	S.E ≤ −2.0 D each eye	38%
Clark and Clark, 2015 [11]	USA	6–15	1.1 ± 0.3	0.01, control	60	−0.25 D < S.E < −8.0D Astigmatism < −2.0 D	75%
Diaz-Llopia and Pinazo-Durán, 2018 [12]	Spain	9–12	5	0.01, control	200	−0.5 D < S.E < −2.0 D Astigmatism < −1.5 D	77%
Yam et al., 2018 [8]	Hong-Kong	4–12	1	0.05, 0.025, 0.01, placebo	383	S.E < −1.0 D Astigmatism < −2.5 D	27%
Moon and Shin, 2018 [10]	Korea	5–15	1	0.05, 0.025, 0.01	285	S.E > −6.0 D Astigmatism < −1.5 D	48%
Joachimsen et al., 2019 [14]	Germany	6–17	1	0.01	56	ΔS.E > 0.5 D/year	62%
Sacchi et al., 2019 [15]	Italy	5–16	1	0.01	102	ΔS.E > 0.5 D/year	55%

RE: Refractive Error. Rate change was calculated as change in S.E. per year. The study follow-up duration mean and SD is shown for the Clark & Clark stusy. LAMP = Yam et al., 2018, ATOM = Chia et al., 2015.

## Data Availability

The data presented in this study are available in the present article.
